# Die Reform des Betreuungsrechts

**DOI:** 10.1007/s00115-022-01355-6

**Published:** 2022-09-14

**Authors:** Tanja Henking

**Affiliations:** Institut für Angewandte Sozialwissenschaften (IFAS), Hochschule für angewandte Wissenschaften Würzburg-Schweinfurt, Münzstr. 12, 97070 Würzburg, Deutschland

**Keywords:** Wunschbefolgung, Erforderlichkeitsgrundsatz, Entscheidungsunterstützung, Ehegattenvertretung, Selbstbestimmung, Consideration of wishes, Necessity principle, Supported decision-making, Representation of spouses, Self-determination

## Abstract

Die Betreuungsrechtsreform ist beschlossen! Zum 01.01.2023 tritt sie in Kraft. Das Betreuungsrecht wird in seiner inhaltlichen Ausrichtung nicht völlig neu aufgestellt. Das Spannungsverhältnis zwischen dem Schutz der Person vor einer nicht eigenverantwortlichen Entscheidung und der Selbstbestimmung der Person wird konsequent weitergedacht und die Selbstbestimmung der betroffenen Person gestärkt. Unterstützende Entscheidungsfindung, die Befolgung von Wünschen und die Aufgabe des Begriffs „Wohl“ als Maßstab für das Betreuerhandeln sind einige Beispiele hierfür. Das neue Recht zieht auch Grenzen bei der Befolgung von Wünschen. Nämlich dann, wenn mit ihnen die Gefahr einer erheblichen Schädigung der Person oder ihres Vermögens einhergeht. Der Beitrag stellt zunächst wesentliche Ziele der Reform dar. Hiervon ausgehend werden Schwerpunkte gesetzt, die für die psychiatrische Praxis relevant erscheinen: die Streichung des Worts „Wohl“, die Regelung der Vorsorgevollmacht, Maßnahmen zur stärkeren Beachtung und zur besseren Umsetzung des im Betreuungsrecht zentralen Grundsatzes der Erforderlichkeit. Kritisch wird Stellung bezogen zum neu eingefügten Ehegattenvertretungsrecht. Im Ergebnis bleibt festzustellen, dass im Bereich der Gesundheitssorge und erst recht im Bereich der Zwangsmaßnahmen für die Praxis recht wenig bedeutsame Änderungen eintreten werden. Ob sich das begrüßenswerte Anliegen des Gesetzgebers verwirklicht, Betreuungen durch eine verbesserte Information und Beratung zu sozialen Rechten und Ansprüchen zu vermeiden, wird die Praxis erst noch zeigen müssen. Gleiches gilt sicherlich auch für eine Stärkung der Entscheidungsunterstützung, eine Idee, die ebenfalls zu begrüßen ist, für die die Praxis aber noch nach einem Goldstandard sucht.

## Abstract (en)

The reform of the guardianship law has been decided! The reform will come into force on 1 January 2023. The content of the guardianship law will not be completely restructured. The tense relationship between the protection of the person against decisions not made on his or her own responsibility and the self-determination of the person will be consistently further considered and strengthened in favor of the self-determination of the person concerned. Supportive decision making, the consideration of a person’s wishes, the abandonment of the term (and concept of) “well-being” as a measure for guardianship are some examples; however, the new law also sets boundaries in adhering to a person’s wishes. Namely, if they are associated with the risk of significant harm to the person or his or her property. The article first presents the main objectives of the reform. Based on this, the focal points are the deletion of the word “well-being”, the regulation of the proxy power of attorney and measures for greater consideration and better implementation of the principle of the necessity, which is central to the guardianship law. Critical comments are made on the newly introduced spousal representation law and its significance for the psychiatric practice. As a result, it can be stated that there will be hardly any significant changes in healthcare and even less so in the area of coercive measures. It remains to be seen whether the legislator’s welcome concern to avoid guardianship through improved information and counselling on social rights will be achieved in practice. The same certainly applies to the strengthening of supported decision making, the idea of which is also to be welcomed but is still looking for a gold standard for practice.

Zum 01.01.2023 tritt die große Reform des Betreuungsrechts in Kraft, die sämtliche Paragrafen durcheinanderwirbelt, sie neu zusammensetzt und eine konsequente Fortführung eines Erwachsenenschutzkonzepts darstellt, das die Selbstbestimmung des Einzelnen beachten will. Der Beitrag gibt einen Überblick über ausgewählte, klinisch relevante Bereiche des reformierten Betreuungsrechts und nimmt dessen Ziele in den Blick. Das neue Recht tritt mit den Zielen Qualitätssteigerung und Mehr an Selbstbestimmung an. Ob diese Ziele mit der Reform erreicht werden, wird der Praxistest der nächsten Jahre zeigen.

## Hintergründe und Ziele der Reform

### I.

Das Betreuungsrecht feiert in diesem Jahr seinen 30. Geburtstag. Das 1992 in Kraft getretene Betreuungsrecht gilt als eine der wichtigsten Reformen des 20. Jahrhunderts [4, 9]. Im kommenden Jahr tritt nun nach einer Übergangszeit, die der Praxis ausreichend Zeit zur Vorbereitung auf die Änderungen geben soll, eine weitere Reform des Betreuungsrechts in Kraft [3]. Die Regelungen zu Zwangsmaßnahmen waren nicht Gegenstand der Reform. Daher werden sie im Folgenden nur Erwähnung finden, soweit sie durch andere ausgewählte Regelungen berührt werden. Eine absolute Neuerung enthält das zukünftige Betreuungsrecht: ein automatisches Ehegattenvertretungsrecht [6]. Dieses von den Bundesländern eingebrachte Konzept stieß und stößt auf einige Bedenken, sodass es später noch einmal einer kritischen Betrachtung unterzogen werden soll.

Eine absolute Neuerung im zukünftigen Betreuungsrecht: ein automatisches Ehegattenvertretungsrecht

Die Reform stellt keine inhaltliche Ablösung vom „alten“ Betreuungsrecht dar. Vielmehr ist sie eine konsequente Umsetzung und Fortführung von bereits im aktuell gültigen Betreuungsrecht geltenden Prinzipien [2, 4, 5, 10, 11]. Es würde der Reform bei Weitem nicht gerecht, sie lediglich als Paragrafenwanderung [12] oder als kosmetische Korrektur zu verstehen. Diese – große – Reform darf als gelungen bezeichnet werden, hält jedoch einige herausfordernde Punkte für die Praxis bereit. An vorderster Stelle sind dabei der Wechsel hin zur Wunschbefolgung, weg von der Wohlschranke [2, 5, 10, 11] und die konsequente(re) Betonung des Erforderlichkeitsgrundsatzes zu nennen. Die Aufgabe, den Schutz der betroffenen Person vor (nicht eigenverantwortlicher) Selbstschädigung und die Anerkennung und Förderung von Selbstbestimmung in Ausgleich zu bringen, ist und bleibt das zentrale Spannungsverhältnis im Betreuungsrecht.

Zur Erinnerung: Das derzeit noch geltende Betreuungsrecht leitete 1992 einen Paradigmenwechsel ein. Die Entmündigung wurde abgeschafft. Wer Betreuung mit Geschäftsunfähigkeit (oder Einwilligungsunfähigkeit) gleichsetzt, unterliegt einem Irrtum. Die Reform des Betreuungsrechts bietet eine gute Gelegenheit, sich diese nach wie vor verbreitete Fehlvorstellung noch einmal ins Bewusstsein zu rufen und Betreuung als ein am tatsächlichen Bedarf ausgerichtetes Schutzkonzept und nicht als Bevormundung oder Fremdbestimmung jeglicher Art zu verstehen. Vielleicht gelingt es so auch, eine sprachliche Sensibilisierung zu erreichen und Formulierungen wie „steht unter Betreuung“ aus dem Wortschatz zu streichen. Der Gesetzgeber hat versucht, eine als diskriminierend empfundene Wortwahl zu vermeiden und spricht zukünftig nicht mehr von psychischer Krankheit oder einer körperlichen, geistigen oder seelischen Behinderung, sondern von Krankheit oder Behinderung, § 1814 Bürgerliches Gesetzbuch (BGB; [3, 10, 11]).

Gleichwohl wird es auch in Zukunft Betreuungen gegen den Willen von Personen geben. Ebenso werden Zwangsmaßnahmen wie medikamentöse Zwangsbehandlung oder Fixierung mit dem neuen Betreuungsrecht weiterhin möglich sein.

### II.

Der Betreuungsrechtsreform ging die Evaluation des Betreuungsrechts mit Blick auf zwei zentrale Fragen des Betreuungsrechts voraus: die Frage nach der Qualität der gesetzlichen Betreuung [7] und die Frage nach der Beachtung respektive Umsetzung des im Gesetz verankerten Erforderlichkeitsgrundsatzes [8]. Der Reformprozess selbst gestaltete sich bemerkenswert partizipativ unter Beteiligung verschiedener Expertinnen und Experten aus unterschiedlichen Bereichen sowie in verschiedener Hinsicht betroffener Personen und Gruppen.

### III.

In der Vergangenheit wurden unterschiedliche Positionen zur Frage vertreten, ob das deutsche Betreuungsrecht den Vorgaben der UN-Behindertenrechtskonvention (UN-BRK) gerecht wird. Die wohl herrschende Lesart war, dass das Betreuungsrecht UN-BRK-konform sei, jedenfalls dann, wenn der Erforderlichkeitsgrundsatz und die Entscheidungsunterstützung ausreichend Beachtung finden. Die gegenteilige Auffassung, die 2015 vom UN-Fachausschuss mit den „Abschließenden Bemerkungen zum ersten Staatenbericht Deutschlands“ vorgetragen wurde, findet in den Rechtswissenschaften wie auch im politischen Diskurs weitgehend keine Zustimmung. Hierauf nimmt auch der Reformgesetzgeber in seinen Begründungen ausdrücklich Bezug und verweist dabei auf die Entscheidungen des Bundesverfassungsgerichts (BVerfG), das sich ausführlich mit Art. 12 UN-BRK auseinandergesetzt hat und aus der Regelung kein absolutes Verbot stellvertretender Entscheidungen ableitet (BVerfGE 142, 313–353; 149, 293–345; [3]). Die Reform nimmt ausdrücklich auf die UN-BRK Bezug und erkennt die von der UN-BRK ausgehenden wichtigen Impulse, betont insbesondere die Selbstbestimmung der betroffenen Person, stellt diese in den Mittelpunkt und verankert im Gesetz verstärkt das Prinzip der Entscheidungsunterstützung, das darauf zielt, der Person das Treffen einer eigenen Entscheidung zu ermöglichen. In § 1821 Abs. 1 S. 2 BGB n. F., der die Aufgaben des Betreuers festlegt, wird ausdrücklich von Unterstützung gesprochen. Zudem wird für das Betreuerhandeln ein Wunschbefolgungsprinzip der Orientierungsmaßstab [2–4, 10, 11].

### IV.

Die Reform verfolgt überdies die Idee, die Rechtsanwendung zu erleichtern [3, 10, 11]. Die Paragrafen haben einen neuen Platz erhalten und sind nach inhaltlichen Zusammenhängen neu sortiert worden [4]. Dies wird die Rechtsanwendung und vor allem die Auffindbarkeit von Vorschriften zukünftig erheblich erleichtern.

### V.

Die Reform spielt sich aber nicht lediglich in den materiellen Voraussetzungen des BGB ab (und auch dort nicht nur im Betreuungsrecht), sondern auch im Verfahrensrecht. An dieser Stelle soll der Hinweis genügen, dass die Änderungen im Verfahrensrecht deutlicher hätten ausfallen können. Zu erwähnen ist noch das neue Betreuungsorganisationsgesetz (BtOG). In § 5 BtOG sind die Aufgaben der Betreuungsbehörde festgelegt: die Information und die Beratung über Unterstützungsmöglichkeiten und andere Hilfen, insbesondere über soziale Rechte. Die Behörde soll zudem gemäß § 8 BtOG dem oder der Betroffenen zur Vermeidung der Bestellung eines Betreuers oder einer Betreuerin ein Beratungs- und Unterstützungsangebot unterbreiten. Das im BtOG vorgesehene Registrierungsverfahren für Berufsbetreuerinnen und -betreuer soll zur Qualitätssteigerung beitragen (§§ 23 ff. BtOG). Das BtOG enthält überdies bereichsspezifische Datenschutzbestimmungen.

## Ausgewählte Bereiche und ihre Konsequenzen für die psychiatrische Praxis

### I. Streichung des Begriffs „Wohl“

Im ersten Moment überraschen die Streichung des Begriffs „Wohl“ aus dem Betreuungsrecht und die neue Pflicht „Wunschbefolgung“ [2–4, 10, 11]. Insbesondere aus einer medizinethischen bzw. ärztlichen Perspektive gehört die Verpflichtung, zum Wohl des Patienten bzw. der Patientin zu handeln, zu den zentralen Prinzipien. Dieses Prinzip wird mit der Betreuungsreform nicht angegriffen. Zum besseren Verständnis mögen zwei Hinweise helfen. Erstens: Es geht um das Innenverhältnis zwischen Betreuer bzw. Betreuerin und betreuter Person. § 1821 BGB n. F. legt die Pflichten des Betreuers oder der Betreuerin fest. Auch nach derzeit noch geltendem Betreuungsrecht hat der Betreuer oder die Betreuerin als Interessensvertreter des bzw. der Betreuten gemäß § 1901 Abs. 2 und 3 BGB dessen bzw. deren Wünsche und Vorstellungen zu befolgen und zu beachten. Klarstellend gilt zu erwähnen, dass dies auch die Wünsche der nicht einwilligungsfähigen bzw. nicht geschäftsfähigen Person betrifft. Begrenzt wird dies durch die Bindung an das Wohl der betreuten Person sowie die Zumutbarkeit für den Betreuer bzw. die Betreuerin [1]. Diese „Wohlschranke“ entfällt zukünftig [3]. Zweitens: Wohl war bereits im noch geltenden Betreuungsrecht aus der Perspektive der betroffenen Person und nicht (rein) objektiv zu verstehen. Dies wurde in der Praxis zuweilen missverstanden [3]. Wohl ist nach der Rechtsprechung des Bundesgerichthofs in Verbindung zu den subjektiven Vorstellungen und Wünschen der betreuten Person zu sehen. Erst wenn die Befolgung von (ernsthaft vorgetragenen) Wünschen höchstrangige Rechtsgüter der betreuten Person gefährdet, ist nach bisherigem Recht die Wohlschranke erreicht [1].

Das Spannungsverhältnis zwischen der Beachtung von Selbstbestimmung und dem Schutz vor nicht eigenverantwortlicher Selbstschädigung bringt das neue Betreuungsrecht nun noch stärker zum Ausdruck [2, 3, 10, 11]. „Wohl“ fällt aus dem Sprachgebrauch des Gesetzes heraus; eine Grenze für die Wunschbefolgungspflicht kennt es dennoch weiterhin. Denn nach § 1821 Abs. 3 BGB n. F. ist der Wunsch nicht zu befolgen, wenn damit eine erhebliche Gefahr für die Person oder das Vermögen der Person einhergeht. Vorausgesetzt, es handelt sich um eine nicht eigenverantwortliche Entscheidung der betreuten Person: Nach § 1821 Abs. 3 Nr. 1 BGB n. F. darf die betreute Person die Gefahr aufgrund ihrer Krankheit oder Behinderung nicht erkennen oder nicht nach dieser Einsicht handeln können. Letztlich steht hinter „Gefahr“ für die Person doch wieder eine Erwägung, ob diese Handlung das Wohl der Person (einschließlich ihres Vermögens) gefährdet [2, 3, 5]. Die Gefährdung selbst muss erheblich sein. Für den Betreuer bzw. die Betreuerin heißt es, zukünftig zu prüfen, ob eine Entscheidung bzw. eine daraus resultierende Handlung zu einer erheblichen Gefährdung der Person (oder ihres Vermögens) führt und ob der Betreute zu dem Zeitpunkt eigenverantwortlich agiert oder nicht. Ebenso wie es für die Rechtsanwendungspraxis eine Klärung durch die Gerichte für die Auslegung von „Wohl“ bedurfte (Stichwort: Wohlschranke; [1]), wird man auch hier eine Klärung und Justierung durch die Gerichte erwarten dürfen [2, 10].

Der Begriff „Wohl“ fällt aus dem Sprachgebrauch des Gesetzes heraus

Insbesondere im Kontext von Handeln gegen den natürlichen Willen und letztlich Zwang zeigt sich, wo die Pflicht zur Wunschverfolgung an ihre Grenzen stößt und staatliche Schutzpflichten dazu aufrufen, Wünsche sogar zu missachten. Konkret dann, wenn die Befolgung von Wünschen zu einer erheblichen gesundheitlichen Selbstschädigung führen würde [5]. Die Regelungen zur Unterbringung, für unterbringungsähnliche Maßnahmen und für die Zwangsbehandlung bleiben inhaltlich unverändert; diese Maßnahmen sind nur zur Abwehr erheblicher gesundheitlicher Schäden bzw. zur Abwendung eines Suizids zulässig.

Keine Veränderung für den Bereich Gesundheitssorge stellt die Pflicht zur Ermittlung des mutmaßlichen Willens dar, wenn der Wunsch weder bekannt noch festzustellen ist und außerdem keine Patientenverfügung vorliegt. Dies sieht § 1821 Abs. 4 BGB n. F. nun grundsätzlich für jedwedes Betreuerhandeln vor. Die Regelung zur Patientenverfügung (§ 1901a BGB, zukünftig § 1827 BGB) bleibt inhaltlich gleich. Die amtliche Überschrift Patientenverfügung wird erweitert um die Begriffe Behandlungswunsch und mutmaßlicher Wille, was jedenfalls für die Anwendungspraxis ein Signal sein dürfte, die Prüfung der Vorgaben des § 1827 BGB nicht bei Fehlen einer (verbindlichen) Patientenverfügung vorschnell zu beenden.

### II. Erforderlichkeitsgrundsatz und andere Hilfe

Der Erforderlichkeitsgrundsatz galt bereits für das jetzige Betreuungsrecht [1]. Es hat sich allerdings gezeigt, dass er in der Praxis nicht ausreichend Beachtung gefunden hat [2, 8]. Daher erfährt er nun eine Stärkung [2]. Der Erforderlichkeitsgrundsatz, ein zentrales Prinzip für das gesamte Betreuungsrecht, sieht zum einem vor, dass andere Hilfen vorrangig zur Betreuerbestellung zu prüfen sind. Eine Betreuerbestellung für „alle Aufgabenbereiche“ ist nicht möglich; die einzelnen Aufgabenbereiche sind jeweils einzeln anzuordnen und zuvor im Hinblick auf ihre Erforderlichkeit zu prüfen. Zum anderen gilt aber auch (weiterhin), dass innerhalb der bestehenden Betreuung nur so viel an rechtlicher Betreuung stattfinden soll, wie auch tatsächlich erforderlich ist. Auch jetzt gilt bereits, dass andere Hilfen vorrangig zu prüfen sind. Adressiert sind damit vor allem soziale Hilfen und insbesondere soziale Rechte. Durch den neuen Gesetzeswortlaut in § 1814 Abs. 3 S. 2 Nr. 2 BGB n. F. wird dieses Vorrangprinzip noch einmal besonders herausgestellt. Flankiert wird es durch entsprechende Regelungen im neuen BtOG [13] und in den Sozialgesetzbüchern, wo dieses Prinzip ebenfalls zum Ausdruck kommt. So haben die Betreuungsbehörden in Zukunft im Vorfeld einer Betreuung der betroffenen Person ein Beratungs- und Unterstützungsangebot zu unterbreiten, ihnen kommt die Pflicht zu, mit Zustimmung der betroffenen Person andere Hilfen sowie den Kontakt zwischen der betroffenen Person und Beratungs- und Unterstützungsangeboten zu vermitteln, § 8 Abs. 1 BtOG.

### III. Selbstbestimmte Vertretung durch Vollmacht

Zu den Zielen des Reformgesetzgebers passt es, die selbstbestimmte Erteilung einer Vollmacht weiter zu stärken. Eine Regelung zur Vollmacht wird sich zukünftig im Gesetz unter § 1820 BGB n. F. finden. Dort sind alle Regelungen zur Vollmacht in einer Norm zusammengefasst. Für die Vollmacht findet sich zukünftig gesammelt in § 1820 Abs. 2 BGB n. F. das Erfordernis einer schriftlichen und ausdrücklichen Vollmachtserteilung für bestimmte Aufgabenbereiche (bisher: Einwilligung in ärztliche Maßnahmen § 1904 Abs. 5 BGB, in freiheitsentziehende Maßnahmen § 1906 Abs. 5 BGB, in ärztliche Zwangsmaßnahmen § 1906a Abs. 5 BGB). Diese Form der Sortierung wird im Übrigen auch bei anderen Komplexen umgesetzt, wie unter anderem bei Genehmigungsvorbehalten. Neu ist die Möglichkeit der Bestellung eines Kontrollbetreuers (§ 1820 Abs. 3 BGB n. F.). Das Gericht kann gemäß § 1820 Abs. 4 BGB n. F. anordnen, dass der Bevollmächtigte die ihm erteilte Vollmacht nicht ausüben darf, und zwar dann, wenn die dringende Gefahr besteht, dass er nicht den Wünschen des Vollmachtgebers entsprechend handelt und dadurch die Person des Vollmachtgebers oder deren Vermögen erheblich gefährdet.

### IV. „Automatische“ Vertretung durch Ehegattenvertretung

Im Widerspruch zum Selbstbestimmungsgedanken steht das Ehegatten(not)vertretungsrecht. Es kann somit als Fremdkörper im ansonsten an der Stärkung des Selbstbestimmungsrechts ausgerichteten neuen Betreuungsrecht empfunden werden. Nicht nur, dass es den Gedanken von Autonomie und eigener Vorsorge für den Notfall entgegensteht, es hat auch das Potenzial, Konflikte in Beziehungen zu bringen. Dieses umstrittene Instrument wird für die psychiatrische Behandlung allerdings (oder daher?) nur teilweise eine Rolle spielen. Bevor auf die Beschränkungen eingegangen wird, soll zunächst diese neue Figur dargestellt werden [6]. § 1358 BGB n. F. ist mit „Gegenseitige Vertretung der Ehegatten in Angelegenheiten der Gesundheitssorge“ überschrieben. Bereits hieraus ergibt sich, dass sich das Vertretungsrecht auf Eheleute und auf den Aufgabenbereich der Gesundheitssorge beschränkt. In diesem Aufgabenbereich sind vier Bereiche der Vertretung aufgezählt:

- Die Einwilligung in bzw. das Untersagen von medizinischen Behandlungen (Untersuchungen, Heilbehandlungen, ärztliche Eingriffe)
- Das Eingehen von Verträgen, die im Zusammenhang mit der Gesundheitssorge stehen (Krankenhausvertrag, Behandlungsvertrag). Für Verträge über Maßnahmen der Rehabilitation und der Pflege findet sich bereits die einschränkende Formulierung „eilige Maßnahmen“.
- Die Einwilligung in freiheitsbeschränkende bzw. -entziehende Maßnahmen wie Fixierungen, Gurte, Bettgitter etc. Auch hier gibt es eine Einschränkung: Das Vertretungsrecht beschränkt sich auf Maßnahmen, die im Einzelfall nicht länger als sechs Wochen dauern.
- Das Geltendmachen von Ansprüchen, die aus Anlass der Erkrankung entstehen.
  § 1358 Abs. 2 BGB n. F. enthält konsequenterweise eine Regelung zur ärztlichen Schweigepflicht, von der die behandelnden Ärztinnen und Ärzte gegenüber dem Ehegatten bzw. der Ehegattin in den oben genannten Fällen befreit werden. Entsprechend dürfen sie ihm bzw. ihr auch Einsicht in die Behandlungsunterlagen geben.

Nachdem § 1358 Abs. 1 BGB nun zunächst ein recht weitreichendes Vertretungsrecht einräumt, folgen in § 1358 Abs. 3 BGB diverse Einschränkungen. Die Einschränkungen beziehen sich zunächst auf die Person des Ehegatten bzw. der Ehegattin, der bzw. die dann als Vertreter bzw. Vertreterin ausscheidet, wenn

- die Eheleute getrennt leben,
- entweder dem behandelnden Arzt bzw. der behandelnden Ärztin oder dem Ehegatten bzw. der Ehegattin bekannt ist, dass der oder die Betroffene eine Vertretung durch seine/ihre Gattin oder seinen/ihren Gatten ablehnt,
- eine Vorsorgevollmacht jemand anderen für den Aufgabenbereich benennt oder
- ein Betreuer bzw. eine Betreuerin für den Aufgabenbereich bestimmt ist.

Hier taucht die erste Schwierigkeit für die Praxis auf: Woher nehmen behandelnde Ärztinnen und Ärzte diese Kenntnisse? Das Gesetz spricht lediglich von „bekannt ist“. Eine Nachforschungspflicht ergibt sich hieraus nicht. Der bzw. die Betroffene scheint also – auch trotz Vorsorge durch eine Vorsorgevollmacht – dem automatischen Vertretungsrecht ausgesetzt zu sein. Einen gewissen Schutz bietet der Kontrollmechanismus des § 1358 Abs. 4 BGB n. F. Danach müssen behandelnde Ärztinnen und Ärzte schriftlich bestätigen, dass die in Abs. 1 aufgeführten Voraussetzungen vorliegen. Hierzu gehört auch, dass der bzw. die Betroffene aufgrund von Krankheit oder Bewusstlosigkeit seine bzw. ihre Angelegenheiten rechtlich nicht selbst besorgen kann. Ebenso muss er bzw. sie schriftlich gegenüber dem vertretenden Ehegatten bzw. der vertretenden Ehegattin mitsamt einer Erklärung, dass die genannten Voraussetzungen vorliegen, bestätigen, dass kein Ausschlussgrund nach Abs. 3 besteht. Eine besondere Qualifikation muss der Arzt bzw. die Ärztin nicht aufweisen, um feststellen zu können, dass die betroffene Person nicht in der Lage ist, ihre Angelegenheit zu besorgen, und (ab) wann dieser Zustand eingetreten ist. Der Gesetzgeber hatte wohl vor allem den für die Praxis unproblematischen Fall der Bewusstlosigkeit vor Augen, es sind aber viele weitere weitaus problematischere Konstellationen denkbar – von Delirzuständen über Demenz bis hin zu Psychosen.

Das Prozedere wirkt kompliziert, verlangt zusätzliche Erklärungen und Dokumentationen

Zum anderen muss der Ehegatte bzw. die Ehegattin, der bzw. die den Ehepartner bzw. die Ehepartnerin vertritt, schriftlich versichern, dass er bzw. sie bisher kein Vertretungsrecht ausgeübt hat und kein Ausschlussgrund nach Abs. 3 vorliegt. Damit muss sich der behandelnde Arzt bzw. die behandelnde Ärztin doch Kenntnis darüber verschaffen; denn er bzw. sie hat sich dies nach dem Wortlaut des Gesetzes vergewissern zu lassen. Sollte er bzw. sie falsche Angaben erhalten, wird man ihm bzw. ihr hieraus aber keinen Vorwurf machen können, da weitergehende Nachforschungspflichten nicht bestehen. Anders dürfte es wiederum dann sein, wenn er bzw. sie Kenntnis von Angaben erhält, die im Widerspruch zu den Angaben des vertretenden Ehegatten bzw. der vertretenden Ehegattin stehen. Man wird hier schnell erkennen: Das Prozedere wirkt kompliziert, es verlangt zusätzliche Erklärungen und Dokumentationen. Für die Praxis lässt sich erahnen, wie dieses nur durchführbar erscheint: mit vorgefertigten Formularen, die zur Unterschrift ohne weitere Erklärungen vorgelegt und zur Akte genommen werden. Dem Schutz der betroffenen Patientinnen und Patienten dürfte in der Praxis nur unzureichend Genüge getan werden. Der hohe Anspruch der ansonsten so gelungenen Reform wird mit dem Ehegattenvertretungsrecht aus vielfältigen Gründen nicht gehalten.

Das Ehegattenvertretungsrecht ist begrenzt auf die ersten sechs Monate nach Eintritt der Vertretungsvoraussetzungen nach Abs. 1, die deshalb eben auch mit Datum zu erfassen sind.

Eine weitere wichtige Begrenzung gilt für den Bereich Psychiatrie. Es darf zwar im Rahmen des Ehegattenvertretungsrechts in (mechanische, medikamentöse oder sonstige) Maßnahmen, die freiheitsentziehend wirken, eingewilligt werden (im Sinne des geltenden § 1906 Abs. 4 BGB, zukünftig § 1831 Abs. 4), nicht jedoch in die Unterbringung oder in die medikamentöse Zwangsmaßnahme/Zwangsbehandlung. Diese Einschränkung ist zu begrüßen. Zu weit geht aber das Vertretungsrecht für freiheitsentziehende Maßnahmen, auch wenn dieses auf sechs Wochen begrenzt wurde. Während dieser Aufgabenkreis im Rahmen einer Vorsorgevollmacht ausdrücklich schriftlich vorgesehen sein muss, ist diese Befugnis nun für sechs Wochen automatisch dem Ehepartner bzw. der Ehepartnerin eingeräumt. Die Forderung nach einem automatischen Ehegattenvertretungsrecht wurde oftmals damit begründet, dass es der Vorstellung vieler Menschen in der Bevölkerung entspreche, dass Eheleute sich gegenseitig im Notfall vertreten würden. Diese ohnehin fragwürdige Annahme für die Gesetzesbegründung wird man bei Entscheidungen gegen den natürlichen Willen – gegebenenfalls unter Anwendung von körperlichem Zwang – nicht unterstellen können. Überdies dürfte es für viele Eheleute eine Überforderung bedeuten, qua eines Ehegattenvertretungsrechts automatisch in eine Entscheidungsverantwortung katapultiert zu werden. Es wird für die Praxis eine Herausforderung werden, die in der Verantwortung stehenden Eheleute bei der Ausübung ihrer Aufgaben zu unterstützen, für diese selbst wird die Praxis Beratungsmöglichkeiten und -zugänge schaffen müssen, soll das Ehegatten(not)vertretungsrecht nicht zu einer übermäßigen Belastung der ohnehin in den Entscheidungssituationen emotional belasteten Ehepartnerinnen und Ehepartnern werden. Um die Rechte der betroffenen, vertretenen Ehepartnerinnen und Ehepartner ausreichend zu schützen, darf das oben genannte Prozedere nicht zu einem reinen Formalismus verkommen, und das Gespräch zur Feststellung des Willens der betroffenen Patientinnen und Patienten 2uss gestärkt werden.

## Fazit für die Praxis

Die zu begrüßende Reform des Betreuungsrechts wird die Praxis vor Herausforderungen stellen. In den Bereichen Gesundheitssorge und Zwangsmaßnahmen werden die Änderungen für die klinische Praxis kaum zu spüren sein. Wenn es aber gelingt, soziale Rechte durch die Betreuungsbehörde frühzeitig und umfassend den von Betreuungsbedarf betroffenen Personen zukommen zu lassen, wäre auch im Hinblick auf die Anwendung von Zwang etwas erreicht. Die bereits oft ausgerufene und überhörte Forderung, frühzeitig Hilfe anzubieten und nicht erst, wenn die Situation eskaliert, würde dann doch Gehör finden. Die Reform verfolgt viele gute Ansätze und erleichtert die Rechtsanwendung durch eine deutlich verbesserte inhaltliche Sortierung der Vorschriften. Die Praxis wird zeigen müssen, ob sie bereit ist, die Ziele anzunehmen, sich den Herausforderungen einer echten Unterstützung statt Stellvertretung zu stellen und diese Unterstützung in der Praxis umzusetzen. Das Ehegattenvertretungsrecht hingegen wird kritisch gesehen und sollte als neues Instrument regelmäßig reflektiert und evaluiert werden.
